# Effectiveness of Traditional and Virtual Surgical Planning in Orthognathic Surgery: A Systematic Review and Meta-Analysis

**DOI:** 10.7759/cureus.79033

**Published:** 2025-02-15

**Authors:** Sumit Gupta

**Affiliations:** 1 Department of Orthodontics, Healthpoint Hospital, Mubadala Health, M42, Abu Dhabi, ARE

**Keywords:** conventional surgical planning, malocclusion, orthognathic surgery, systematic review, traditional surgical planning, virtual surgical planning

## Abstract

Orthognathic surgery, which corrects jaw deformities, requires meticulous planning to achieve optimal functional and aesthetic outcomes. Traditional surgical planning (TSP) relies on manual methods, whereas virtual surgical planning (VSP) uses computer-assisted simulations that may enhance accuracy and efficiency. Therefore, we conducted a systematic review to evaluate the effectiveness of VSP compared to TSP for orthognathic surgery. We adhered to the Preferred Reporting Items for Systematic Reviews and Meta-Analyses guidelines and registered the protocol in the Prospective Register of Systematic Reviews (CRD42024618614). We searched electronic databases for studies comparing VSP and TSP. Two independent reviewers performed screening and data extraction. We assessed quality using the Cochrane Risk of Bias 2 (RoB 2) tool. We calculated the standardized mean difference (SMD) as the summary measure and used a random-effects model, considering p < 0.05 as statistically significant. We performed analyses using Review Manager (RevMan) version 5.3 (Cochrane Collaboration, London, UK). We included 10 qualitative synthesis and meta-analysis studies, evaluating data from 474 patients, of whom 244 underwent TSP and 230 underwent VSP. Included studies showed moderate to low risk of bias. We evaluated effectiveness in terms of planning time, surgical time, horizontal measurements, vertical measurements, and accuracy indicators for predicting outcomes. Meta-analysis revealed reduced planning time (SMD = 3.19 (-5.74 to 0.64)) and surgical time (SMD = -0.42 (-1.32 to 0.49)) with VSP, as well as differences in horizontal (SMD = -0.39 (-0.73 to 0.05)) and vertical measurements (SMD = -0.20 (-0.54 to 0.13)), and in accuracy indicators (SNA0: SMD = -0.15 (-0.46 to 0.16); SNB0: SMD = 0.53 (-0.82 to 1.87)). SNA0 represents the angle formed by sella (S), nasion (N), and point A (A), and SNB0 represents the angle formed by sella (S), nasion (N), and point B (B). The funnel plot showed no evidence of publication bias. VSP reduced planning and surgical times and predicted facial outcomes more accurately than TSP. These findings suggest that VSP can be an effective alternative to TSP in orthognathic surgeries.

## Introduction and background

Orthognathic surgery, the correction of jaw deformities, relies on meticulous preoperative planning, precise intraoperative execution, and careful postoperative care to achieve optimal patient outcomes [1]. Over the past six decades, this surgical specialty has evolved significantly and has become safer, faster, and more effective [2]. The success of these procedures relies on thorough preparation, accurate operative plan implementation, and the prevention of postoperative relapse. A carefully designed approach ensures the correct diagnosis, comprehensive facial analysis, and precise execution of the surgical intervention, ultimately enhancing both functional and aesthetic outcomes [3].

Preoperative planning remains the most critical step in orthognathic surgery [4]. Traditional surgical planning (TSP), which often involves manual model surgery, photography, and two-dimensional radiography, has been the conventional approach for many years. However, TSP methods are limited by complexity, susceptibility to errors, and inability to accurately control the third dimension of jaw and facial structures [5]. Virtual surgical planning (VSP) has emerged as an alternative, offering computer-aided simulations and three-dimensional (3D) representations of the facial skeleton, soft tissue, and dentition [6]. By providing a platform for virtual diagnosis, surgical simulation, and the comparison of multiple treatment approaches, VSP facilitates enhanced decision-making and can improve postoperative outcomes [7].

Comparing VSP with TSP is challenging due to variations in reported outcomes and the diverse methodologies across studies. While VSP often demonstrates superior accuracy in spatial planning, questions remain regarding its cost-effectiveness and long-term clinical applicability [3]. These considerations are particularly relevant when planning complex procedures such as bimaxillary osteotomy, where accurate surgical movements and reliable postoperative stability are critical [5]. When evaluating bimaxillary osteotomy planning methods, factors such as planning time, surgical time, and the accuracy of horizontal and vertical measurements are crucial determinants of success [8]. The overall time required for planning, surgery, and post-treatment follow-up is an important indicator of the efficiency and practicality of VSP and TSP in clinical settings [2,6-8].

Shorter operating times can reduce anesthetic exposure and blood loss, while shorter planning times can streamline patient care and improve resource allocation [9]. Efficient treatment workflows may influence total costs, patient satisfaction, and the feasibility of widespread clinical implementation. VSP offers a comprehensive 3D view of osteotomies and enables soft tissue prediction, which can inform the choice of surgical maneuvers and improve the likelihood of achieving desired outcomes. Although both VSP and TSP can produce acceptable clinical results, current evidence is limited and has not conclusively demonstrated that VSP achieves similar accuracy across all parameters [10]. We updated our research on related literature and conducted a systematic review to provide updated evidence on the effectiveness of VSP compared to TSP. We hypothesized that VSP might be an ideal alternative to TSP in orthognathic surgery, improving efficiency and accuracy while potentially reducing overall treatment times.

## Review

This systematic review was designed according to the Preferred Reporting Items for Systematic Reviews and Meta-Analyses (PRISMA) 2020 checklist [11]. It was registered in the Prospective Register of Systematic Reviews (PROSPERO) with the registration number CRD42024618614. We formulated a focused research question according to the participants (P), intervention (I), comparison (C), and outcome (O) format as follows: “Is there any difference in the effectiveness of VSP compared to TSP for orthognathic surgery?” The "participants" included patients undergoing orthognathic surgery, the "intervention" was VSP, the "comparison" was TSP, and the "outcomes" included planning time, surgical time, horizontal and vertical measurements, and accuracy indicators. Keywords and Medical Subject Headings terms were selected and combined with Boolean operators such as AND/OR, and we conducted the search strategy according to the PICO (population, intervention, comparator, and outcome assessed) format (Table 1).

**Table 1:** PICO format. PICO: population, intervention, comparator, and outcome assessed; MeSH: Medical Subject Headings; 3D: three-dimensional; 2D: two-dimensional.

Component	Strategy
Population	"Facial asymmetry" [MeSH Terms] OR "malocclusion" OR "class II malocclusion" OR ("class III malocclusion" [MeSH Terms] OR "double jaw surgery" OR "orthognathic surgery"
Intervention	("virtual surgical planning" [MeSH Terms] OR ("le fort 1" AND "bilateral sagittal split osteotomy" AND "3D surgical planning" OR "mandibular osteotomy" OR "surgical time" OR ("operating time" [MeSH Terms] OR ("doctor time") [MeSH Terms]
Comparator	("conventional surgical planning" OR "traditional surgical planning" [MeSH Terms] OR ("2D surgical planning" OR "mandibular osteotomy" OR "surgical time" OR ("operating time" [MeSH Terms] OR ("doctor time") [MeSH Terms]
Outcome assessed	("planning time" [MeSH Terms] OR "surgical time" OR ("horizontal measurement" [MeSH Terms] OR ("vertical measurement") AND "bimaxillary osteotomy” OR "orthognathic surgery" OR ("retrospective study") AND "randomized controlled trial" AND "prospective study")

We included studies published in English from January 2000 to September 2024 from open-access sources, provided they contained sufficient comparative data on the effects of VSP and TSP on orthognathic surgery. These studies reported outcomes such as planning time, surgical time, horizontal and vertical measurements, and accuracy indicators and were designed as randomized controlled trials, retrospective studies, or prospective studies that met our predefined criteria [3-5]. We excluded studies conducted before 2000, those not published in English, and those not from open-access sources. We also excluded reviews, abstracts, letters to the editor, editorials, animal studies, and in vitro studies to maintain a focus on direct clinical comparisons.

Screening process

We performed a comprehensive database search until September 2024, covering 24 years, using PubMed, Google Scholar, and EBSCOhost. Two authors conducted a rigorous two-phase screening process, initially evaluating titles and abstracts to remove irrelevant records and assessing full-text articles for eligibility. Disagreements were resolved through discussion, and when necessary, a third reviewer was consulted to achieve consensus. For each included study, we extracted descriptive data, including author information, country of the study, year of publication, sample size, software used for VSP, the type of malocclusion, surgical procedures, outcome measures, follow-up duration, and the authors’ conclusions. To assess methodological quality and risk of bias, we used the Cochrane Collaboration Risk of Bias 2 (RoB 2) tool [12], evaluating several domains within Review Manager (RevMan) version 5.3 software (Cochrane Collaboration, London, UK) to ensure a systematic appraisal of evidence quality.

Statistical analysis

We conducted the statistical analysis using standardized mean difference (SMD) as the summary measure to compare outcomes between VSP and TSP. We considered a p-value less than 0.05 indicative of statistical significance [13]. We employed Cochran’s test for heterogeneity to evaluate the variability in treatment effect estimations among the included trials, deeming heterogeneity statistically significant if the p-value was less than 0.01 [14]. We used Begg’s funnel plot to assess publication bias by plotting effect size against standard error. If the funnel plot appeared asymmetrical, we considered the potential for publication bias and its implications for interpreting the results [15].

Results

**Figure 1:**
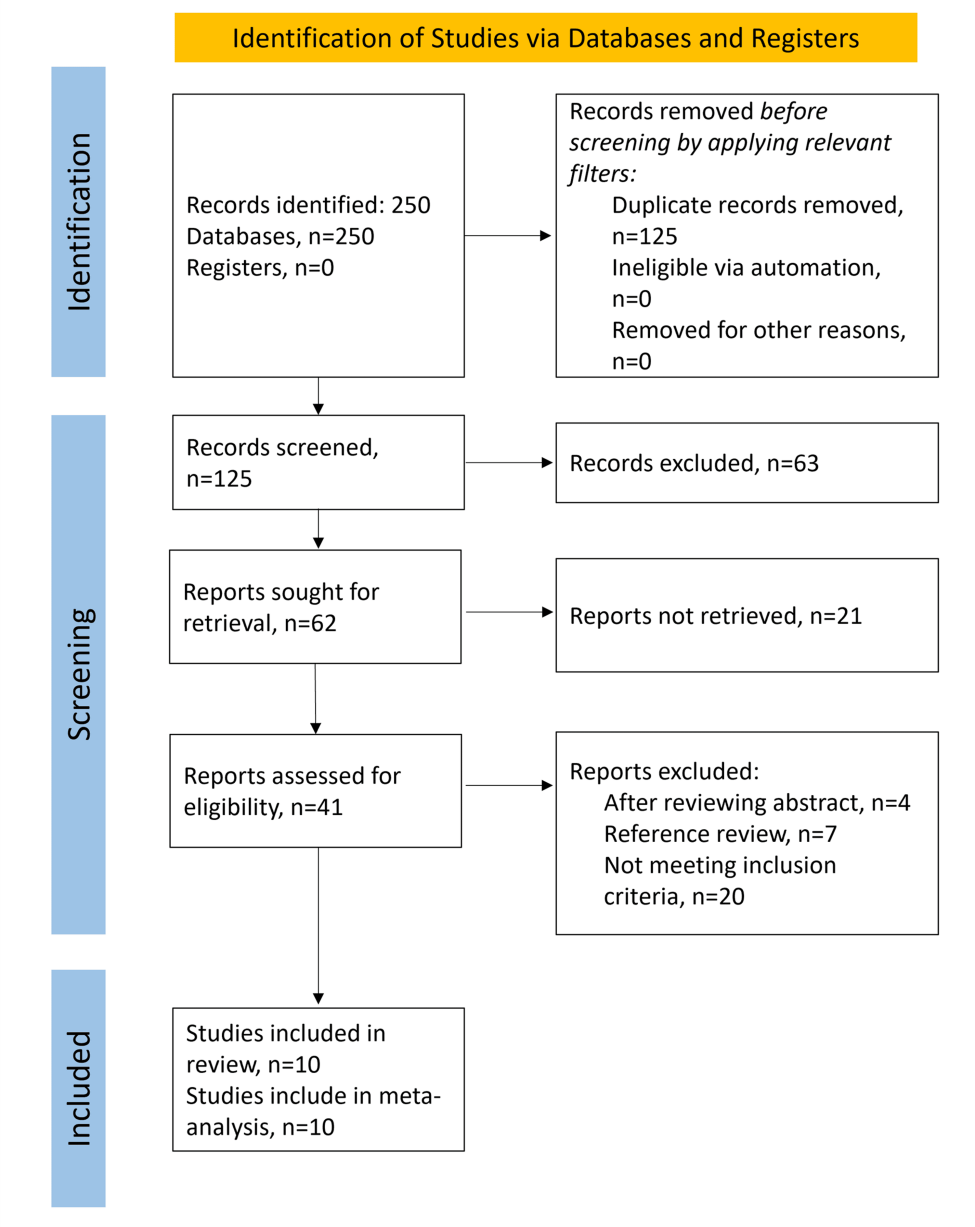
PRISMA 2020 flow diagram. PRISMA: Preferred Reporting Items for Systematic Reviews and Meta-Analyses.

A total of 250 studies were retrieved from the database searches, and after removing duplicates and applying all inclusion and exclusion criteria, 10 studies remained eligible for both qualitative and quantitative synthesis, as illustrated in Figure 1. These studies included 474 patients, of whom 244 were evaluated with TSP and 230 were evaluated with VSP for orthognathic surgery.

As shown in Table 2, the data were drawn from 10 studies [16-25] that all employed a randomized controlled trial (RCT) design. The included studies were conducted in multiple countries, specifically two studies in Korea [16,25], three studies in the United States of America (USA) [17,19,20], and one study each in Belgium [18], Brazil [21], Austria [22], Sweden [23], and Germany [24]. Various computer software programs, including 3Txer (Orapix Co., Seoul, South Korea), 3D Surgery (Dolphin Imaging and Management Solutions, Chatsworth CA, USA), Maxilim (Medicim, Mechelen, Belgium), and Simplant Pro™ (Dentsply, Mölndal, Sweden), were used for VSP. Patients presented with diagnoses such as facial asymmetry and class II and III malocclusions, and they underwent procedures that included bimaxillary orthognathic surgery, mono-maxillary surgery, Le Fort I osteotomy, bilateral sagittal split osteotomy, osteotomy, and genioplasty. We evaluated the effectiveness between TSP and VSP by assessing linear measurements from cephalometry and cone-beam computed tomography images and comparing planning time and surgical time. The results of these studies demonstrated that VSP reduced both planning and surgical time and provided improved accuracy in soft tissue diagnosis and in predicting facial outcomes.

**Table 2:** Descriptive study details of included studies. BSSO: bilateral sagittal split osteotomy; CASS: computer-aided surgical simulation; CBCT: cone-beam computed tomography; LFI: Le Fort I osteotomy; TSP: traditional surgical planning; VSP: virtual surgical planning; SNA0: the angle formed by sella, nasion, and point A; SNB0: the angle formed by sella, nasion, and point B.

Author, year	Country	Sample size, n	Software used	Malocclusion diagnosis	Surgery	Outcomes evaluated	Follow-up duration	Conclusion
Kwon et al. (2014) [16]	Korea	TSP: 23, VSP: 19	3Txer	Facial asymmetry	Bimaxillary	Linear measurements from cephalogram	1 month before to 3 to 5 days after	Reduced surgical time and greater efficiency were seen with VSP
Schwartz (2014) [17]	USA	TSP: 30, VSP: 30	CASS	Class II, III	Bimaxillary orthognathic surgery	Planning and surgical time	Before and after surgery	Surgical time is reduced in VSP
Van Hemelen et al. (2015) [18]	Belgium	TSP: 35, VSP: 31	Maxilim	TSP – class II, VSP – class III	Mono-maxillary	Linear measurements from cephalogram and CBCT images	1 week before to 4 months after	VSP is more accurate in soft tissue planning
Resnick et al. (2016) [19]	USA	TSP: 23, VSP: 20	3D Surgery	Facial asymmetry	Bimaxillary orthognathic surgery	Planning time, soft tissue accuracy	Before to 6 months post surgery	VSP takes less time and is more efficient than TSP
Wrzosek et al. (2016) [20]	USA	TSP: 41, VSP: 41	Not mentioned	Facial asymmetry, class III	Bimaxillary orthognathic surgery	Planning time, surgical time	Before to 12 months post-surgery	The time needed for treatment planning is reduced in VSP
Ritto et al. (2018) [21]	Brazil	TSP: 30, VSP: 30	3D Surgery	Class III	Bimaxillary	Linear measurements from cephalogram and CBCT images	1 week before to 10 days after	Both the techniques had shown equal accuracy (p > 0.05)
Steinhuber et al. (2018) [22]	Austria	TSP: 11, VSP: 11	3D Surgery	Class III	Bimaxillary orthognathic surgery	Working time	Before to 4 months post surgery	The time required for planning was equal in both techniques
Bengtsson et al. (2019) [23]	Sweden	TSP: 29, VSP: 29	Simplant	Class III	BSSO, osteotomy, genioplasty	Planning and surgical time	Before 12 months after surgery	Both techniques have equal accuracy in predicting facial outcomes
Schneider et al. (2019) [24]	Germany	TSP: 12, VSP: 9	3D Surgery	Facial asymmetry	LFI, BSSO	Measurement between pre- and postoperative condition (SNA0/SNB0) and surgery timing	Before to after surgery	Reduction in operation duration with improved accuracy seen with VSP
Park et al. (2021) [25]	Korea	TSP: 10, VSP: 10	Not reported	Facial asymmetry	LFI, BSSO	Planning time, surgical time	Planning time, surgical time	Time invested in VSP was less than TSP

Quality assessment

**Figure 2:**
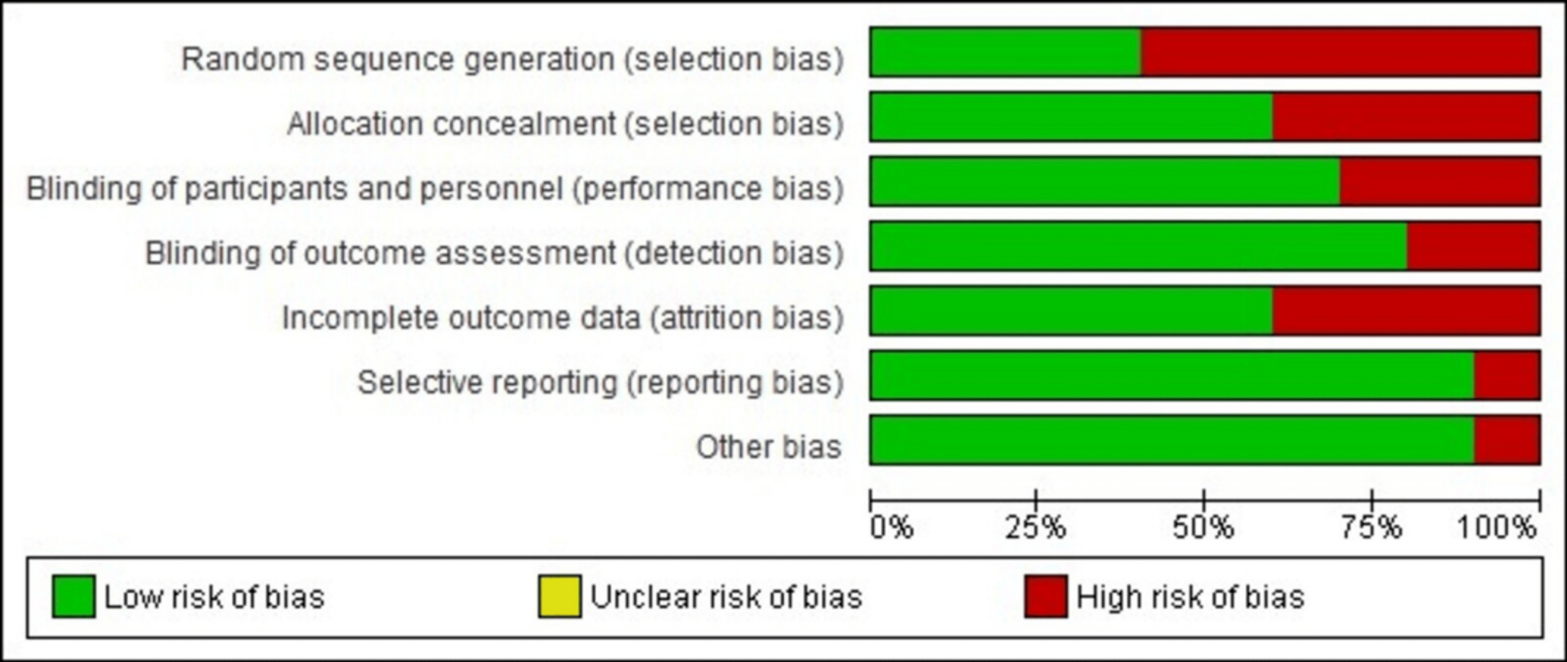
Cochrane risk of bias as percentages across all included studies.

**Figure 3:**
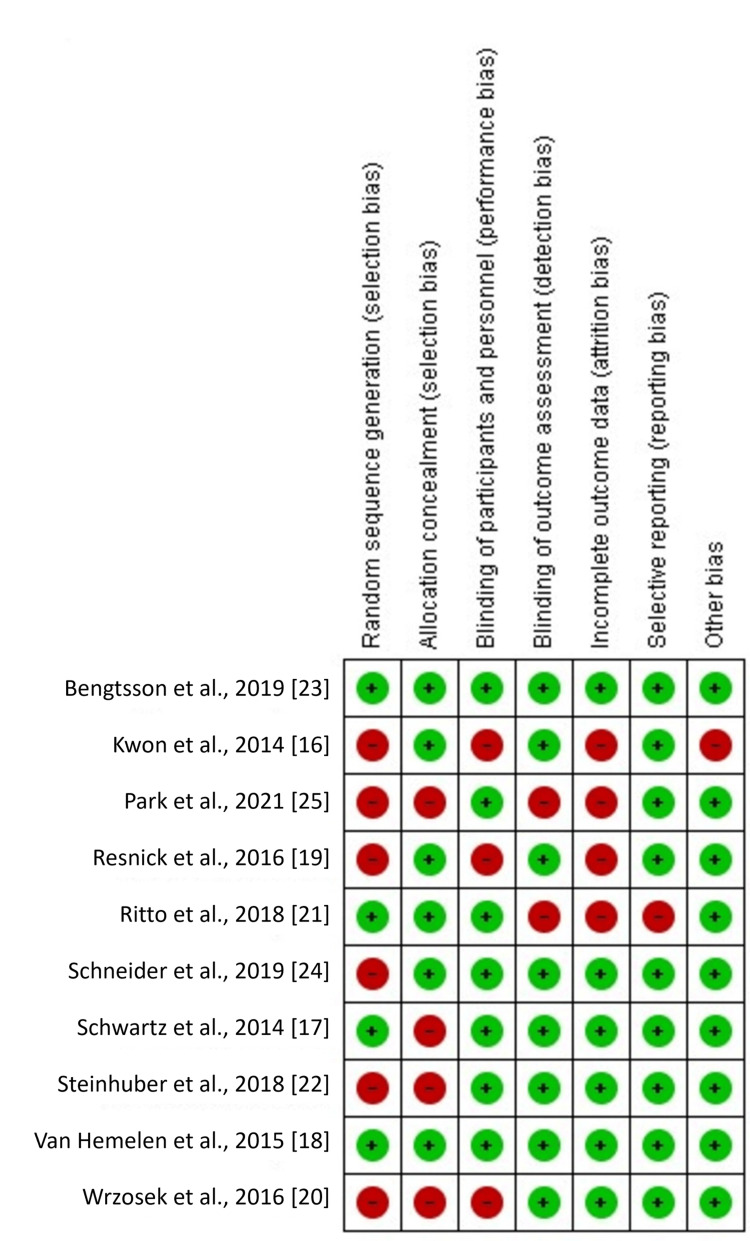
Cochrane risk of bias summary for each study.

Quality assessment was performed using the Cochrane Collaboration’s approach to evaluate the risk of bias (ROB). High ROB was observed for random sequence generation and incomplete outcome data. Although these domains indicated some methodological concerns, all included studies reported moderate to low ROB overall. Domains related to allocation concealment, masking of participants and personnel, masking of outcome assessment, selective reporting, and other sources of bias showed the lowest ROB, as depicted in Figures 2, 3.

Synthesis of results and meta-analysis

**Figure 4:**
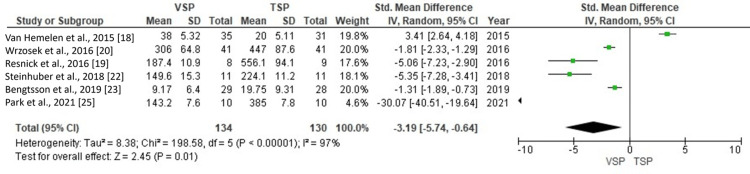
Comparison between VSP and TSP for planning time. VSP: virtual surgical planning; TSP: traditional surgical planning; SD: standard deviation; CI: confidence interval.

**Figure 5:**
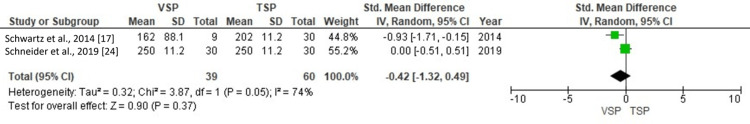
Comparison between VSP and TSP for surgical time. VSP: virtual surgical planning; TSP: traditional surgical planning; SD: standard deviation; CI: confidence interval.

**Figure 6:**
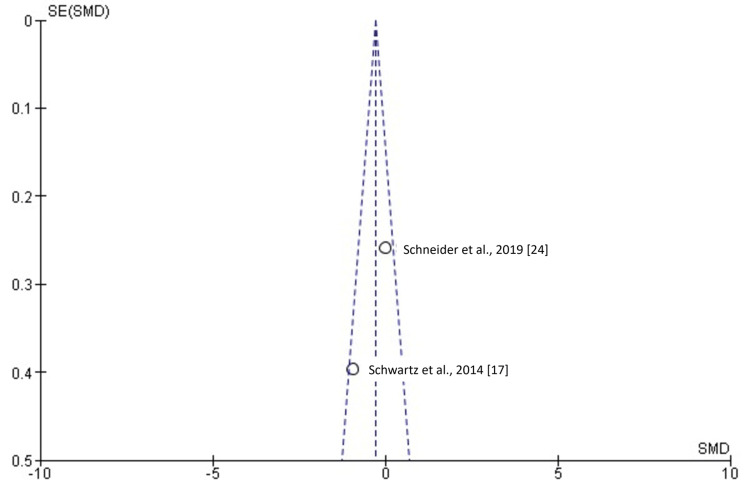
Funnel plot showing the absence of possible publication bias. SMD: standardized mean difference; SE: standard error.

**Figure 7:**
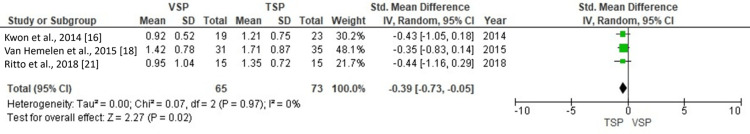
Comparison between VSP and TSP for horizontal measurements. VSP: virtual surgical planning; TSP: traditional surgical planning; SD: standard deviation; CI: confidence interval.

**Figure 8:**
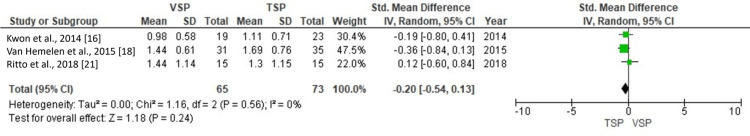
Comparison between VSP and TSP for vertical measurements. VSP: virtual surgical planning; TSP: traditional surgical planning; SD: standard deviation; CI: confidence interval.

**Figure 9:**
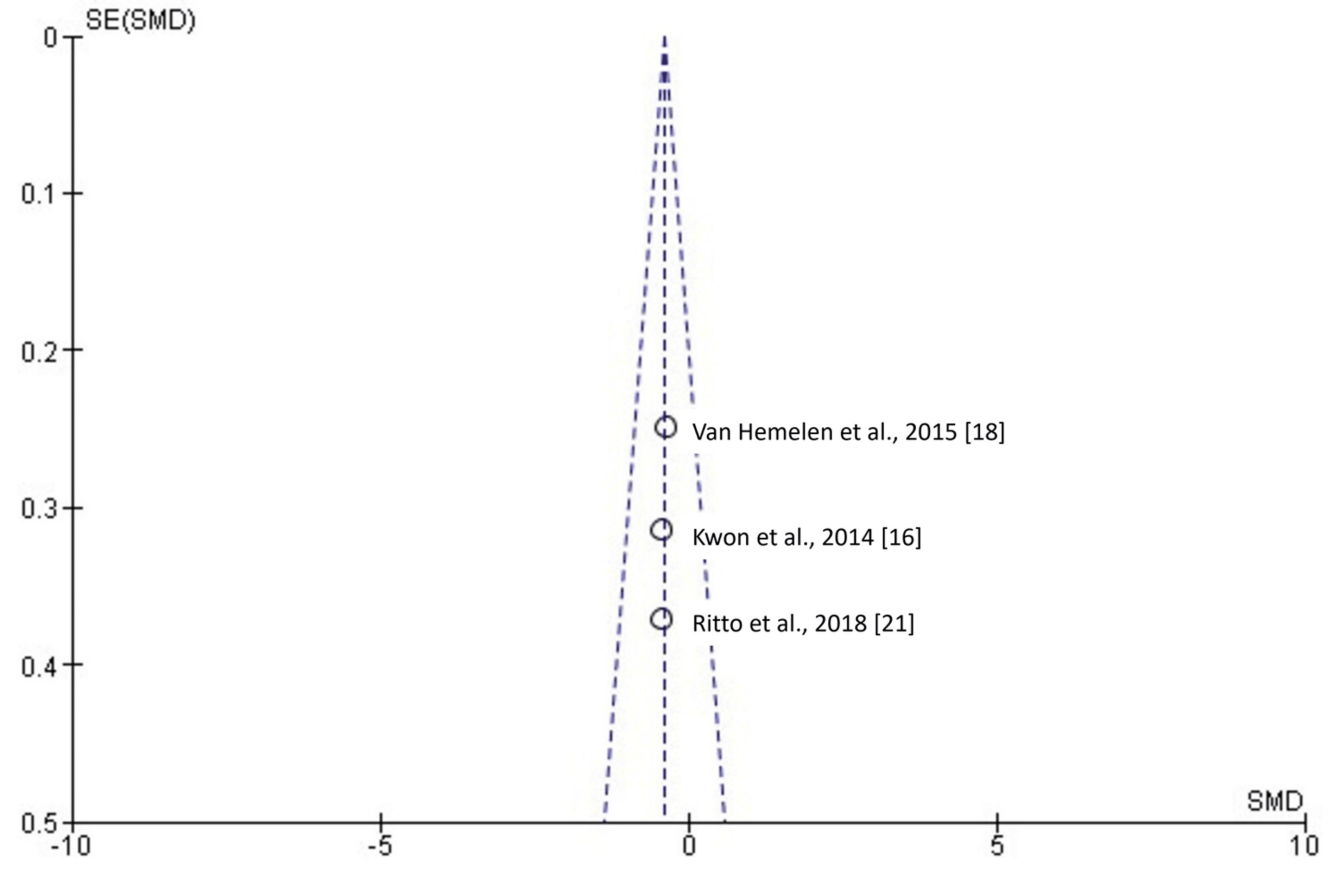
Funnel plot showing the absence of possible publication bias. SMD: standardized mean difference; SE: standard error.

**Figure 10:**
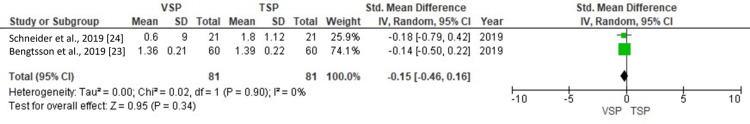
Comparison between VSP and TSP for SNA0. VSP: virtual surgical planning; TSP: traditional surgical planning; SD: standard deviation; CI: confidence interval; SNA0: the angle formed by sella, nasion, and point A.

**Figure 11:**
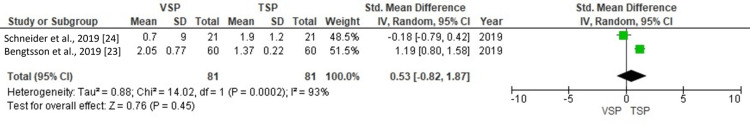
Comparison between VSP and TSP for SNB0. VSP: virtual surgical planning; TSP: traditional surgical planning; SD: standard deviation; CI: confidence interval; SNB0: the angle formed by sella, nasion, and point B.

**Figure 12:**
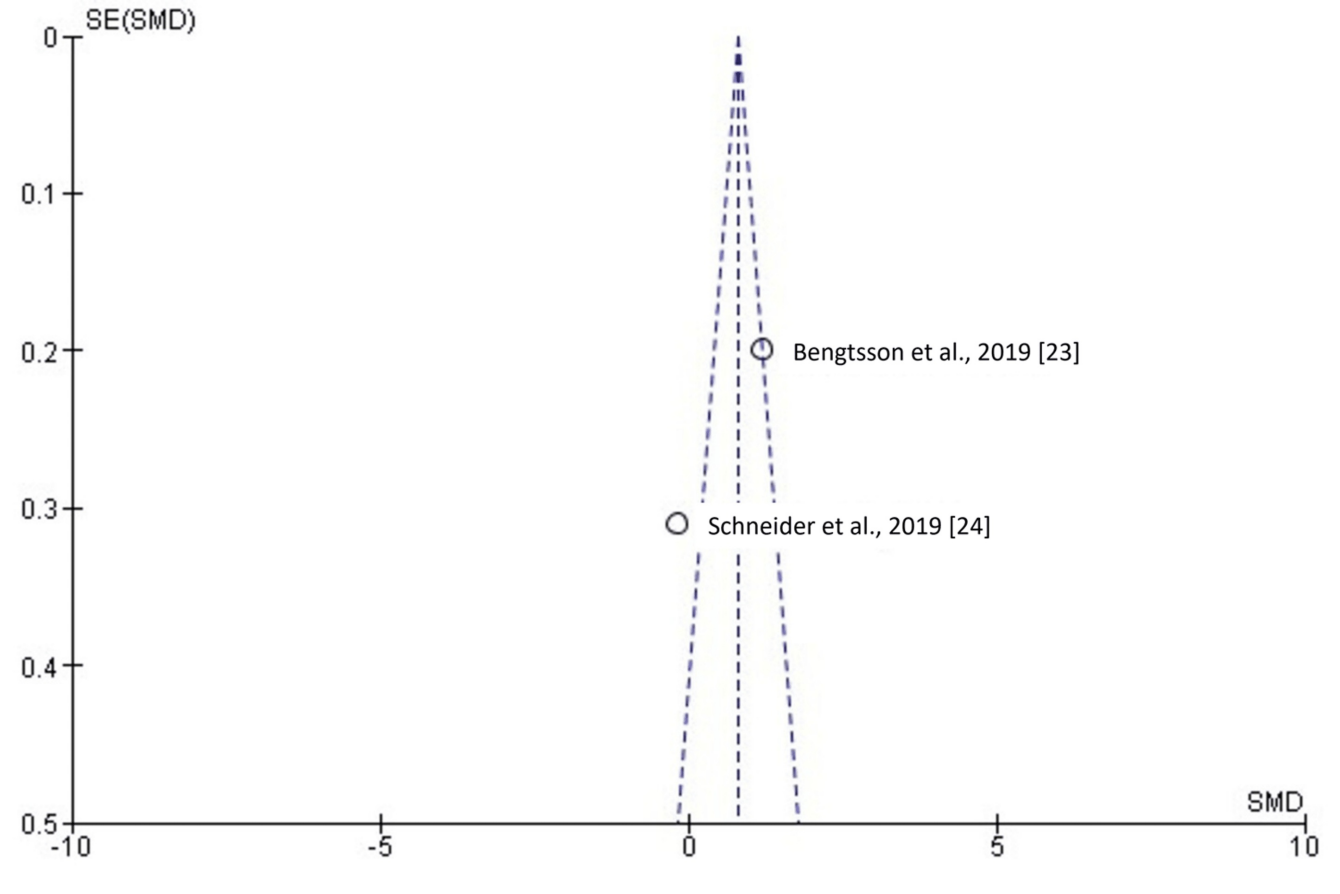
Funnel plot showing the absence of publication bias. SMD: standardized mean difference; SE: standard error.

The effectiveness of VSP and TSP was evaluated by comparing planning time, surgical time, horizontal measurements, vertical measurements, and indicators for predicting surgical accuracy and outcomes, as shown in Figures 4-12. Six studies [18-20,22,23,25] that included 264 patients, with 134 assessed by VSP and 130 by TSP, evaluated planning time. As shown in Figure 4, the SMD was -3.19 (-5.74 to 0.64), indicating that decreased planning time was 3.19 times greater in VSP, and this difference was statistically significant (p < 0.05).

Two studies [17,24] involving 69 patients, of whom 39 were evaluated by VSP and 30 by TSP, examined surgical time. As shown in Figure 5, the SMD was -0.42 (-1.32 to 0.49), suggesting that reduced surgical time was 0.42 times greater in VSP, although this difference was not statistically significant (p > 0.05). Three studies [16,18,21] involving 138 patients, with 65 in the VSP group and 73 in the TSP group, examined horizontal measurements. As shown in Figure 7, the SMD was -0.39 (-0.73 to 0.05), revealing a statistically significant difference (p < 0.05). Three studies [16,18,21] involving 138 patients, with 65 in the VSP group and 73 in the TSP group, examined vertical measurements. As shown in Figure 8, the SMD was -0.20 (-0.54 to 0.13), indicating no statistically significant difference (p > 0.05).

Two cephalometric angles, SNA0 and SNB0, were assessed to evaluate surgical accuracy and predict outcomes. SNA0 represents the angle formed by sella, nasion, and point A, and SNB0 represents the angle formed by sella, nasion, and point B. Two studies [23,24], including 162 patients, with 81 evaluated by VSP and 81 by TSP, analyzed SNA0 measurements. As shown in Figure 10, the SMD for SNA0 was -0.15 (-0.46 to 0.16), indicating no statistically significant difference (p > 0.05). Similarly, two studies [23,24], including 162 patients, 81 in the VSP group and 81 in the TSP group, examined SNB0 measurements. As shown in Figure 11, the SMD was 0.53 (-0.82 to 1.87), and this difference was not statistically significant (p > 0.05).

Discussion

VSP has emerged as a viable alternative to TSP for orthognathic surgery [5]. This approach allows for virtual diagnosis and surgical simulation, enabling surgeons to evaluate multiple treatment plans and potentially optimize postoperative outcomes [6]. Although comparing VSP and TSP is challenging due to variability in reported outcomes across studies, investigating the time required for planning, operation, and the entire treatment process remains critical for determining the clinical effectiveness of these methods [7,8]. Although VSP and TSP both yield acceptable results in certain contexts, there is limited evidence supporting similar accuracy between the two techniques [9,10].

Several previous studies have attempted to address these differences in outcomes. Nilsson et al. [26] conducted a systematic review to evaluate differences in operative and planning times between computer-assisted surgery (CAS) and conventional or traditional planning in cranio-maxillofacial surgery. After reviewing data from 28 studies involving 536 patients in the CAS group and 784 patients in the traditional planning group, they concluded that CAS reduced planning time preoperatively. Chen et al. [27] performed a systematic review and meta-analysis of RCTs comparing VSP and TSP regarding hard and soft tissue accuracy and time requirements. Their analysis of five RCTs, including 199 patients, suggested that VSP improved both hard and soft tissue accuracy in the sagittal plane and reduced overall operative time, concluding that VSP can serve as a good alternative to TSP.

Further supporting these findings, Alkaabi et al. [28] conducted a systematic review and meta-analysis examining the effectiveness of VSP versus TSP in orthognathic surgery or bimaxillary osteotomy. They evaluated eight studies qualitatively and six studies quantitatively, showing that VSP had superior planning time compared to TSP, with a weighted mean difference (WMD) of -5.29 (confidence interval (CI) = -7.90 to 2.68) and greater overall time efficiency (WMD = -0.10 (CI = -0.51 to 0.34)). Similarly, Strujak et al. [29] conducted a systematic review and meta-analysis comparing the accuracy of maxillary and mandibular movements when planned virtually or using conventional techniques. Their results, drawn from six studies (two RCTs and four retrospective studies), including 255 patients, demonstrated that VSP achieved better accuracy for mandibular transverse linear measurements and maxillary horizontal plane measurements, showing that VSP can provide greater precision than TSP.

Despite the availability of these systematic reviews and meta-analyses [26-29], data heterogeneity often hindered the formation of comprehensive qualitative and quantitative comparisons of VSP and TSP for orthognathic surgery. According to our knowledge, this is the first systematic review and meta-analysis to evaluate and compare these two techniques' overall effectiveness while adhering to strict methodological standards. We searched multiple databases for RCTs, retrospective, and prospective studies published from January 2000 to September 2024 that compared VSP and TSP in orthognathic surgery, ultimately including 10 studies [16-25] for qualitative and quantitative synthesis. The included studies generally had a low risk of bias and reported outcomes related to planning time, surgical time, horizontal and vertical measurements, and specific indicators of surgical accuracy and predictive outcomes, as shown in Figures 4-12.

Our meta-analysis revealed that VSP reduced planning time by an SMD of 3.19 (-5.74 to 0.64) and surgical time by an SMD of -0.42 (-1.32 to 0.49) while also demonstrating improvements in horizontal measurements, vertical measurements, and cephalometric indicators such as SNA0 and SNB0 angles. Although some findings did not reach statistical significance, the overall trend suggested that VSP was more time-efficient and accurate than TSP. The funnel plot showed no publication bias, and the systematic review followed the PRISMA guidelines and used the Cochrane RoB 2 tool, ensuring robust methodology and transparent reporting.

Systematic reviews and meta-analyses represent the highest level of evidence in evidence-based practice because they provide transparent, reproducible methodologies and synthesize findings from multiple studies. However, the quality and heterogeneity of included studies can influence the strength and applicability of the evidence. Although our review included studies characterized by brief observation periods and known risks of bias, the overall quality of the included data was sufficient to provide meaningful insights into the comparative effectiveness of VSP and TSP in orthognathic surgery.

## Conclusions

This systematic review and meta-analysis examined whether VSP is a superior alternative to TSP in orthognathic surgery. We investigated differences in planning time, surgical time, and accuracy of predicting soft tissue changes and facial outcomes. Our findings demonstrated that VSP significantly reduced planning and surgical time and improved accuracy in soft tissue diagnosis, yielding more predictable facial outcomes. These results indicate that VSP is clinically and statistically superior to TSP, highlighting its potential to streamline treatment workflows, improve patient care, and enhance surgical precision. Our findings underscore the importance of integrating VSP into routine practice, with implications for better resource utilization, reduced procedural times, and improved patient satisfaction in orthognathic surgery.
